# Congenital Thrombocytosis, Hepatosplenomegaly, and Rash in a Term Neonate

**DOI:** 10.1055/a-2851-8535

**Published:** 2026-04-29

**Authors:** Michal Mia Shalamov, Akshara Vadyala, Nirzar Parikh, Jessica Scerbo, Estephanie Rivero

**Affiliations:** 1 23498 Department of Pediatrics, HMH K. Hovnanian Children’s Hospital at Jersey Shore University Medical Center, Neptune City, NJ, United States; 2 23498 Department of Pediatrics, Division of Neonatology, HMH K. Hovnanian Children’s Hospital at Jersey Shore University Medical Center, Neptune City, NJ, United States; 3 23498 Department of Pediatrics, Division of Hematology/Oncology, HMH K. Hovnanian Children’s Hospital at Jersey Shore University Medical Center, Neptune City, NJ, United States

**Keywords:** transient abnormal myelopoiesis, mosaic Down syndrome, GATA1, blast cells, thrombocytosis, hepatosplenomegaly

## Abstract

**Objective** Transient abnormal myelopoiesis (TAM) is a self-limited clonal myeloproliferative disorder seen almost exclusively in neonates with trisomy 21 and defined by circulating myeloblasts carrying N-terminal truncating GATA1 mutations. Although most cases occur in infants with typical Down syndrome features, TAM can arise in clinically normal newborns with mosaic trisomy 21, creating significant diagnostic uncertainty.

**Study Design** We report a term female neonate who presented with pallor, respiratory distress, hepatosplenomegaly, and a papular, nonblanching rash.

**Results** Laboratory studies showed marked thrombocytosis, leukocytosis, and numerous circulating blasts. Flow cytometry detected a 17% abnormal blast population resembling congenital acute myeloid leukemia, but bone marrow aspirate revealed a myeloproliferative picture without definitive malignancy, favoring TAM. Molecular testing confirmed a truncating GATA1 mutation and mosaic trisomy 21 by SNP array, fluorescence in situ hybridization, and microarray. The infant’s condition improved rapidly, with resolution of organomegaly and normalization of blood counts in the first week of life.

**Conclusion** This case underscores the diagnostic challenges of TAM in phenotypically normal infants. Because clinical and laboratory findings can closely mimic congenital leukemia, early evaluation for GATA1 mutations and trisomy 21 is essential to establish the correct diagnosis, guide management, and avoid unnecessary chemotherapy.

## Key Points

- TAM can occur in neonates with mosaic Down syndrome, even in the absence of classic phenotypic features or associated comorbidities.
- TAM should be included in the differential diagnosis of neonates presenting with findings suggestive of congenital leukemia.
- Early recognition requires careful physical examination combined with timely GATA1 mutation analysis.
- Molecular confirmation allows accurate diagnosis, preventing unnecessary intensive chemotherapy and guiding conservative, targeted management.

## Introduction

A term female neonate presents at birth with thrombocytosis, hepatosplenomegaly, and a papular erythematous rash over the cheeks.

Prenatal and birth histories:

- Born to a 38-year-old gravida 3, para 2 woman with Hashimoto’s Thyroiditis.
- Prenatal maternal serologies unremarkable except for positive Group B Streptococcus.
- Prenatal course notable for fetal macrosomia on ultrasound. Maternal serum alpha-fetoprotein screening and genetic testing, including noninvasive prenatal testing, were normal and low risk, respectively.
- Maternal medications: Ibuprofen, Levothyroxine, prenatal multivitamins, and low-dose aspirin.
- Delivered at 39 ^1/7^ weeks of gestation by urgent cesarean section for decreased fetal heart rate variability.
- Resuscitation included noninvasive respiratory support. Apgar scores are 3 and 8 at 1 and 5 min, respectively.

## Presentation after Birth

At birth, the infant was pale and cyanotic, requiring positive pressure ventilation and continuous positive airway pressure, and was admitted to the Special Care Nursery. The abdomen was distended with a palpable liver edge. Empiric antibiotic therapy with ampicillin and gentamicin was initiated. A routine complete blood count (CBC) revealed marked thrombocytosis (platelets 999 × 10 ^3^ /μL), and peripheral smear examination showed numerous blasts, raising concern for a possible hematologic disorder. A repeat CBC demonstrated further platelet elevation, reaching 1079 × 10 ^3^ /μL.

**Fig. 1:**
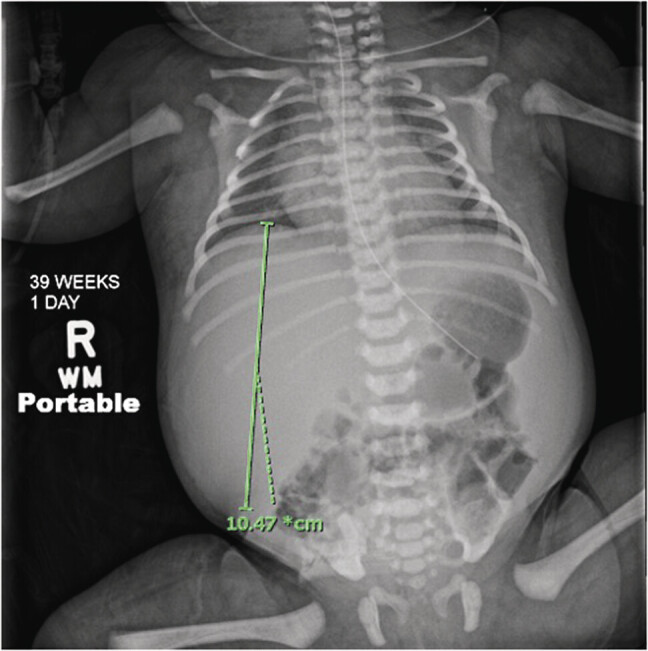
Abdominal X-ray image taken on DOL 0 showing liver at 10.5 cm in the mid-axillary line and splenic enlargement.

Chest and abdominal radiographs demonstrated hepatosplenomegaly ( Fig. 1 ). The Hematology-Oncology team was consulted, and the infant was transferred to a local children’s hospital for further evaluation.

Vital signs

- Heart rate: 136 beats/min.
- Respiratory rate: 48 breaths/min.
- Axillary temperature: 97.5°F (36.4 °C).
- BP: 70/38 mmHg.
- Oxygen saturation: 98%.

Physical examination

**Fig. 2:**
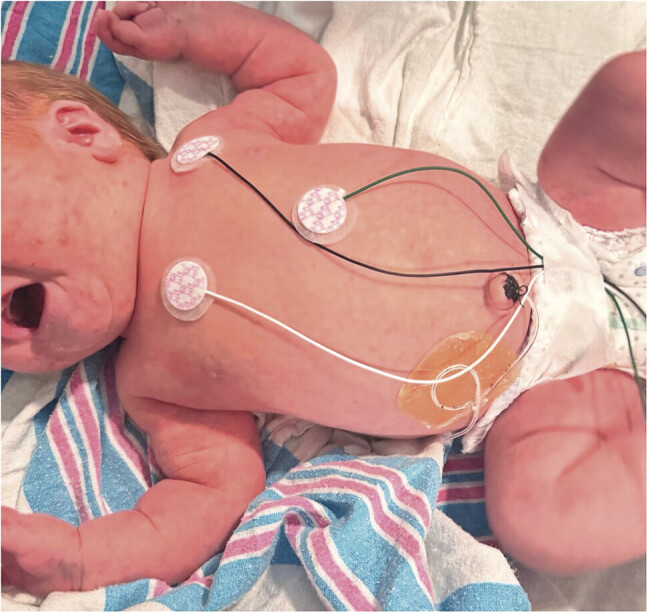
Image of newborn taken on DOL 0 showing scattered papular erythematous nonblanching rash on cheeks, arms, abdomen, and legs.

- Infant was large for gestational age with birth weight: 4250 g (97%), birth length: 50 cm (67%), and head circumference: 36.5 cm (98%).
- General: No acute distress.
- Head: Anterior and posterior fontanelles open and flat.
- Lungs: Clear, equal breath sounds bilaterally; no increased work of breathing.
- Cardiovascular: Normal heart sounds; regular rate and rhythm; no murmurs or gallops.
- Abdomen: Abdominal distension, palpable liver edge, and spleen nontender, normal bowel sounds, 3 vessel cord intact.
- Genitourinary: Normal female genitalia with patent anus.
- Skeletal: Hips normal, extremities without deformity, spine intact without deformity.
- Neurological: Awake and alert, appropriate tone and primitive reflexes.
- Skin: Papular, erythematous, nonblanching rash distributed across the cheeks, arms, abdomen, and legs ( Fig. 2 ).

Laboratory studies

- Platelets: 999 × 10 ^3^ /μL (999 × 109/L), Hemoglobin: 12.7 mg/dL, White blood cells: 26.1 × 10 ^3^ /μL (26.1 × 10 ^9^ /L).
- Peripheral smear: Thrombocytosis, Leukocytosis with numerous, large circulating blasts.

## Differential Diagnosis

- Congenital leukemia.
- Early onset sepsis.
- Stress marrow response.
- Congenital Syphilis.
- Transient abnormal myelopoiesis.

## Actual Diagnosis

Transient abnormal myelopoiesis (TAM) with mosaic Down syndrome.

## Clinical Course, Evaluation, and Management

The infant was transferred from an outside facility on day of life (DOL) 1. She was weaned to room air on DOL 1 before transfer and remained hemodynamically and clinically stable upon arrival. Given the presence of rash, hepatosplenomegaly, thrombocytosis, and circulating blasts, there was concern for possible leukemia or TAM. TAM was initially considered less likely because of the infant’s nonsyndromic appearance and normal prenatal screening results. Reactive thrombocytosis secondary to infection was also considered unlikely, given the absence of clinical signs of sepsis and the presence of circulating blasts.

Nonetheless, the infant was continued on empiric antibiotic therapy for possible sepsis, and flow cytometry was performed on DOL 1. In the meantime, daily laboratory monitoring was performed to assess for tumor lysis syndrome and thrombocytosis, including lactate dehydrogenase (LDH), uric acid, magnesium, phosphorus, and CBC every 12 h, as well as serial abdominal exams. LDH peaked at 1334 U/L before gradually declining. The platelet count reached a maximum of 1079 × 10 ^9^ /L and subsequently trended downward. The infant experienced one episode of prolonged bleeding from the umbilical line; coagulation studies demonstrated a PT of 13.4 s, INR of 1.2, PTT of 39 s, and fibrinogen of 255 mg/dL, with no evidence of coagulopathy.

Peripheral flow cytometry analysis resulted in DOL 4, revealing a 17% abnormal blast population with immunophenotypic features consistent with abnormal myeloblasts. The differential diagnosis included acute myeloid leukemia (AML; favored) versus stress myelopoiesis (less likely). Patient remained on room air, hemodynamically and clinically stable, because peripheral flow cytometry analysis revealed blasts <25%, and the patient continued to demonstrate persistently abnormal peripheral blood counts. A decision was made to perform a bone marrow biopsy on DOL 4 to evaluate for acute congenital leukemias. Concurrently, to expedite diagnosis, genetic testing was performed on DOL 4, including bone marrow fluorescence in situ hybridization (FISH) and chromosomal analysis, as well as oligo SNP chromosomal microarray of peripheral blood.

**Fig. 3:**
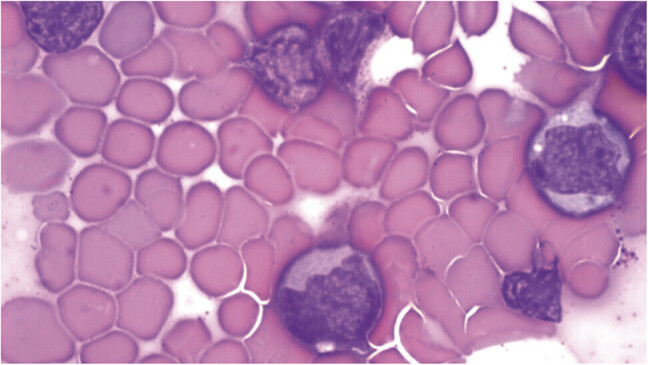
Bone marrow aspirate of left iliac crest showing myeloid blasts.

By DOL 7, the infant demonstrated improved thrombocytosis, resolved leukocytosis, stable anemia, and decreasing hepatosplenomegaly. Bone marrow aspirate resulted in DOL 5, revealing 3.4% myeloblasts ( Fig. 3 ) without morphologic evidence of malignancy. The patient was discharged at DOL 7 with close Hematology-Oncology follow-up.

Genetic testing results became available postdischarge on DOL 14. The peripheral blood oligo SNP array demonstrated an abnormal pattern consistent with mosaic trisomy 21. Bone marrow FISH was positive for gain of the RUNX1 region, and chromosomal analysis found an abnormal karyotype with trisomy 21. In addition, a low-level somatic GATA1 mutation was detected on Genomic Hematology PLUS bone marrow analysis—present in 5.8% of DNA and 97.3% of RNA—supporting the diagnosis of TAM associated with mosaic Down syndrome. Peripheral blood and bone marrow analysis, coupled with resolving blasts, confirmed the diagnosis of TAM.

## Discussion

TAM is a clonal hematopoietic disorder that occurs almost exclusively in neonates with trisomy 21. It is characterized by circulating blasts harboring N-terminal truncating mutations in the GATA1 gene. 1 2 Clinical presentations range from incidental hematologic abnormalities—such as cytopenias and circulating blasts—to severe, life-threatening systemic involvement from disseminated leukemic infiltration. 3

The present case demonstrates several hallmark features of TAM: circulating myeloblast-like blasts, thrombocytopenia, hepatosplenomegaly, and a papulovesicular facial rash that evolved into impetiginous crusts. The combination of a rash resembling impetigo, thrombocytopenia, and hepatosplenomegaly prompted consideration of a hematologic disorder.

Cutaneous manifestations of TAM can resemble those seen in other neonatal leukemias. This presentation parallels prior reports by Shivamallappa et al. 4 5 and Krawczyk et al., 6 who described phenotypically normal neonates with GATA1-positive mosaic trisomy 21 and vesiculopustular or bullous eruptions that resolved following low-dose cytarabine therapy. Similarly, Narvaez-Rosales et al. reported four male infants with papulopustular or vesicular rashes and hepatomegaly, three of whom demonstrated an M7 immunophenotype consistent with TAM. 7

Nakamura et al noted a strong positive correlation between blast count and platelet count at diagnosis, suggesting that thrombocytosis in TAM may reflect increased platelet production by the abnormal megakaryocytic clone rather than normal thrombopoiesis. Elevated platelet or blast counts at presentation may predict prolonged thrombocytopenia and delayed platelet recovery. 8

In this case, the level of thrombocytosis correlated with blast percentage, with maximal platelets of 1,008,000/μL and 20% blasts in peripheral blood on DOL 2. We then observed a steady decline of both cell populations in the next 3 weeks, with the infant achieving a normal platelet count (157,000/μL) and no blasts identified on peripheral smear on DOL 19. While thrombocytosis is less common, when present at diagnosis, it is typically transient. More commonly, an inverse correlation occurs due to the GATA1 mutation in hematopoietic cells, leading to the proliferation of megakaryoblasts, thereby leading to thrombocytopenia. In addition, the recovery phase typically begins at 2–4 weeks of age, with full resolution by 8–12 weeks. In our case, these phases occurred far sooner, suggesting the patient may have begun the recovery phase prenatally, therefore explaining the presence of thrombocytosis.

Although our patient lacked phenotypic features of Down syndrome, FISH confirmed mosaic trisomy 21, and GATA1 sequencing established the diagnosis of TAM rather than AML. Similar cases have been reported by Prudowsky et al., Williams et al., Kawase et al., and Richards et al., describing phenotypically normal neonates with mosaic Down syndrome confirmed by cytogenetic evidence of trisomy 21, RUNX1 or GATA1 analysis, and clinical resolution following supportive or low-dose cytarabine therapy. 9 10 11 12

Recognition of this constellation—transient myeloblastemia, hepatosplenomegaly, cutaneous lesions, and GATA1 positivity—is critical for timely diagnosis and conservative management. Most cases of TAM resolve spontaneously or with brief courses of low-dose cytarabine, underscoring the importance of distinguishing TAM from congenital leukemia to avoid overtreatment.

Although the initial presentation raised concern for AML—particularly acute megakaryoblastic leukemia—this diagnosis is inconsistent with the patient’s spontaneous clinical improvement and transient hematologic abnormalities. AML typically demonstrates progressive blast accumulation, marrow failure, and requires multi-agent chemotherapy, whereas TAM resolves spontaneously within 3 months. 3 9 The presence of a GATA1 truncating mutation and mosaic trisomy 21, as reported in similar cases by Kawase et al 10 and Richards et al, 12 definitively distinguishes TAM from de novo AML or stress-induced marrow responses, in which GATA1 mutations are absent.

Infectious etiologies, including bullous impetigo, congenital syphilis, and viral exanthems, were also excluded based on the presence of circulating blasts, leukemoid reaction, and hepatosplenomegaly—features not seen in isolated infections. 13 Additionally, congenital leukemia cutis may mimic TAM dermatologically but lacks GATA1 mutations and does not resolve without aggressive chemotherapy. 4 7 14

Taken together, the transient clinical course, molecular signature (GATA1 mutation combined with trisomy 21), and resolution with minimal therapy confirm TAM as the most accurate diagnosis in this patient.

## References

[1] Prudowsky Z Han H Stevens A Transient abnormal myelopoeisis and mosaic down syndrome in a phenotypically normal newborn Children 2020 7 06 52 32481622 10.3390/children7060052 PMC7346181

[2] Bhatnagar N Nizery L Tunstall O Vyas P Roberts I Transient abnormal myelopoiesis and AML in down syndrome: an update Curr Hematol Malig Rep 2016 11 05 333 341 27510823 10.1007/s11899-016-0338-x PMC5031718

[3] Williams B A Meyn M S Hitzler J K Transient leukemia in newborns without down syndrome: diagnostic and management challenges J Pediatr Hematol Oncol 2011 33 06 e261 e263 21768885 10.1097/MPH.0b013e3182159f4e

[4] Shivamallappa M D Mullins A Browning Carmo K Bullous eruptions in transient abnormal myelopoiesis with normal phenotype BMJ Case Rep 2023 16 04 e251523 10.1136/bcr-2022-251523 PMC10083739 37028822

[5] Nijhawan A Baselga E Gonzalez-Ensenat M A Vesiculopustular eruptions in down syndrome neonates with myeloproliferative disorders Arch Dermatol 2001 137 06 760 763 11405767

[6] Krawczyk J McDermott M Irvine A D O’Marcaigh A Storey L Smith O Skin involvement in down syndrome transient abnormal myelopoiesis Br J Haematol 2012 157 03 280 22385123 10.1111/j.1365-2141.2012.09079.x

[7] Narvaez-Rosales V de-Ocariz M S Carrasco-Daza D Neonatal vesiculopustular eruption associated with transient myeloproliferative disorder: report of four cases Int J Dermatol 2013 52 10 1202 1209 23046498 10.1111/j.1365-4632.2012.05501.x

[8] Nakamura W Goto H Hayashi A Factors influencing platelet normalization of transient abnormal myelopoiesis Pediatr Int 2020 62 08 907 910 32124502 10.1111/ped.14214

[9] Wu X Sulavik D Roulston D Lim M S Spontaneous remission of congenital acute myeloid leukemia with t(8;16)(p11;13) Pediatr Blood Cancer 2011 56 02 331 332 21157904 10.1002/pbc.22859

[10] Kawase K Azuma E Ohshita H Risk factors for early death in transient myeloproliferative disorder without phenotypic features of down syndrome: a case report and literature review J Pediatr Hematol Oncol 2012 34 06 475 479 22510770 10.1097/MPH.0b013e318249597f

[11] Inaba H Londero M Maurer S H Acute megakaryoblastic leukemia without GATA1 mutation after transient myeloproliferative disorder in an infant without down syndrome J Clin Oncol 2011 29 09 e230 e233 21205752 10.1200/JCO.2010.32.3634 PMC3527733

[12] Richards M Welch J Watmore A Readett D Vora A J Trisomy 21 associated transient neonatal myeloproliferation in the absence of down’s syndrome Arch Dis Child Fetal Neonatal Ed 1998 79 03 F215 F217 10194996 10.1136/fn.79.3.f215 PMC1720867

[13] Heymann W R Carder K R Brown S Neonatal dermatology J Am Acad Dermatol 2004 51 02 287 288 15280849 10.1016/j.jaad.2004.04.016

[14] Solky B A Yang F C Xu X Levins P Transient myeloproliferative disorder causing a vesiculopustular eruption in a phenotypically normal neonate Pediatr Dermatol 2004 21 05 551 554 15461760 10.1111/j.0736-8046.2004.21505.x

